# Comparison of efficacy between subcutaneous and intravenous application of moss‐aGal in the mouse model of Fabry disease

**DOI:** 10.1002/jmd2.12393

**Published:** 2023-09-12

**Authors:** Paulina Dabrowska‐Schlepp, Andreas Busch, Jin‐Song Shen, Rachel Y. Cheong, Lone Bruhn Madsen, Daniel Mascher, Raphael Schiffmann, Andreas Schaaf

**Affiliations:** ^1^ Eleva GmbH Freiburg Germany; ^2^ Institute of Metabolic Disease, Baylor Scott & White Research Institute Dallas Texas USA; ^3^ Scantox Sweden (Previously Timeline Bioresearch AB) Lund Sweden; ^4^ pharm‐analyt Labor GmbH Baden Austria

**Keywords:** enzyme replacement therapy, Fabry disease, moss‐aGal, subcutaneous application

## Abstract

Fabry disease (FD, OMIM 301500) is a rare X‐linked inherited lysosomal storage disorder associated with reduced activities of α‐galactosidase A (aGal, EC 3.2.1.22). The current standard of care for FD is based on enzyme replacement therapy (ERT), in which a recombinantly produced version of αGal is intravenously (iv) applied to Fabry patients in biweekly intervals. Though the iv application is clinically efficacious, periodical infusions are inconvenient, time‐ and resource‐consuming and they negatively impact the patients’ quality of life. Subcutaneous (sc) injection, in contrast, is an established route of administration for treatment of chronic conditions. It opens the beneficial option of self‐administration, thereby improving patients’ quality of life and at the same time reducing treatment costs. We have previously shown that Moss‐α‐Galactosidase (moss‐aGal), recombinantly produced in the moss *Physcomitrium patens*, is efficient in degrading accumulated Gb3 in target organs of murine model of FD and in the phase I clinical study, we obtained first efficacy evidence in human patients following single iv infusion. Here, we tested the efficacy of subcutaneous administration of moss‐aGal and compared it with the results observed following iv infusion in Fabry mice. The obtained findings demonstrate that subcutaneously applied moss‐aGal is correctly transported to target organs and efficacious in degrading Gb3 deposits there and thus suggest the possibility of using this route of administration for therapy of Fabry disease.

Synopsis

Subcutaneously applied moss‐aGal is correctly transported to target organs and principally efficacious in degrading Gb3 deposits in the murine model of Fabry disease.

## 1 INTRODUCTION

Fabry disease (FD, OMIM 301500) is a rare X‐linked lysosomal storage disorder associated with reduced activities of α‐galactosidase A (αGal, EC 3.2.1.22). Mutations in the αGal encoding gene (*GLA*) lead to deficiency of the enzyme, normally responsible for cleavage of terminal galactose moieties from sphingolipids (globotriaosylaceramide Gb3). Dysfunction of α‐Gal causes accumulation of unprocessed lipids in lysosomes and in turn leads to cell damage resulting in renal impairment, cardiomyopathy, and cerebrovascular events.^1^

The current standard of care for FD is an enzyme replacement therapy (ERT), in which recombinantly produced version of αGal: Agalsidase alfa (Replagal) or Agalsidase beta (Fabrazyme) is intravenously (iv) administered to patients in biweekly intervals. Though the iv administration is clinically efficacious,^2^ it is associated with several drawbacks. The infusions are time‐ and resource‐consuming.^3^, ^4^ With short plasma (<120 min) and tissue half‐lives (24–48 h) of both marketed products,^5^, ^6^, ^7^ there is a substantial period between the infusions where enzyme concentration is absent. Infusions are also associated with unwanted reactions.^8^ Although in several countries ERT treatments are realized at private homes,^9^, ^10^ the infusions still constitute a burden in patients’ lives. In 2017, an additional therapy option ‐ orally applied chaperon therapy with Migalastat (Galafold) was approved for a limited group of patients with specific, amenable mutations.^1^ Recently, Pegunigalsidase alfa (Elfabrio), a modified version of αGal, with prolonged half‐life^11^ was approved in Europe and in USA.^12^, ^13^

An alternative route of administration via a subcutaneous (sc) injection is common in chronic conditions (e.g., insulin).^14^, ^15^ Sc injections can be self‐administered in regular intervals and substances applied this way are absorbed slower than iv injections, leading to prolonged exposition in plasma.^16^ In recent years, increase of volume for the sc application could be facilitated with hyaluronidase (rHuPH20, Halozyme^17^). Since 2006 several protein biotherapeutics (e.g., antibodies) have been approved for sc delivery.^18^ Besides patient benefits frequent sc administration could lead to a more constant αGal‐level in circulation, as opposed to biweekly iv infusions.

Previously, we showed that Moss‐α‐Galactosidase (moss‐aGal) was efficient in degrading accumulated Gb3 in target organs of the Fabry mice.^19^ Similarly, a decrease of Gb3 and lyso‐Gb3 levels in urine and plasma was observed following a single iv infusion of moss‐aGal in human patients.^20^ Recognizing the possibility for improvement of patients' life quality, we here tested whether a subcutaneous administration of moss‐aGal proves efficacious in reducing Gb3 deposits in the murine model of FD.

## 2 MATERIALS AND METHODS

### 2.1 Production and purification of moss‐aGal

Moss‐aGal was produced in the moss *Physcomitrium patens* as described previously.^19^ Moss‐aGal was provided in 20 mM Tris, 300 mM NaCl, pH 7.0 for PK and tissue distribution studies and in 20 mM Tris, 250 mM sorbitol, 0.02% Tween, pH 7.0 for efficacy studies (0.8–1.2 mg/mL). Moss‐aGal was diluted with formulation buffer to corresponding dose concentrations. For the cellular localization study, a formulation with approx. 6 mg/mL was used.

### 2.2 PK study

Eight to 11 months old male Fabry mice as described in Ref. 19 were used. For the sc administration, moss‐aGal (200 μL) was injected in the skin between shoulders at 1, 3 or 10 mg/kg body weight (bw) (*n* = 5). Blood was collected at 0.5, 1, 2‐, 4‐, 6‐ and 24‐hours post‐injection by tail bleed. For the iv‐administration, moss‐aGal was injected via the tail vein at a dose of 1 mg/kg body weight (*n* = 5). Blood samples were collected at 1, 5‐, 10‐, 20‐ and 30‐min post injection. Plasma from untreated Fabry mice was used for baseline activity. α‐Gal activity in plasma was measured using 4MU method, as previously described.^19^

### 2.3 Tissue distribution study

About 24 h after injection, mice applied in the PK study were perfused with saline; heart, kidney and liver were dissected, and stored at <−80°C until use. Tissues were homogenized in ice‐cold saline containing 0.2% Triton, and α‐Gal activity was measured.

### 2.4 Short time tissue distribution study

Approximately 5 months old female homozygous Fabry mice were used. Moss‐aGal was diluted in saline to a total volume of 200 μL per mouse and injected subcutaneously at 1 and 3 mg/kg bw (*n* = 5). About 4 h post injection, heart, kidney, and liver were harvested as described above. For the iv‐administration, moss‐aGal was injected as described above and organ harvest was performed 2 h post injection. Untreated wild type mice and Fabry mice were included as controls.

### 2.5 Cellular localization study

Moss‐aGal was injected subcutaneously (200 μL) into 2–3 months old male Fabry mice at 35 mg/kg bw (*n* = 3) and for iv‐administration via tail‐vein at 5 mg/kg bw (*n* = 3). For mock control, the formulation buffer was injected (*n* = 1). At 4 h post‐injection, mice were fixed via transcardial perfusion of formaldehyde, and heart, kidney, and liver were dissected and post‐fixed in the same fixative. After histology processing, tissues were embedded in paraffin and 5 μm sections were cut. α‐Gal was detected by immunohistochemistry using α‐Gal‐specific rabbit polyclonal antibody (Sigma). The signal was detected by DAB as chromogen, and hematoxylin was used for counterstaining. The pictures were taken under 40× lens.

### 2.6 Efficacy studies

Fabry mice (B6;129‐Gla<tm1Kul>/J, Stock Number: 003535) were obtained from Jackson Laboratory. Female homozygous mice at 11 weeks were used, together with age‐matched female wild type animals.

The first efficacy study was based on 4 groups with Fabry mice, and not treated WT group (*n* = 5). Three Fabry mice groups received 8 doses of moss‐aGal (1,3,10 mg/kg bw) subcutaneously, every 2–3 days, over 17 days. The control group was treated with vehicle. The main study was performed with three groups of Fabry mice, and not treated WT group (*n* = 5). The sc group received 16 doses of moss‐aGal (3 mg/kg bw), every 2–3 days, over 36 days. The iv‐group received three doses of moss‐aGal (1 mg/kg bw) on day 1, 15 and 29 via the tail vein. The third control group received vehicle treatment, with a dosing regimen consistent with the sc treated group. All treated animals were dosed at a dosing volume of 8.4 mL/kg.

A terminal blood sample for lyso‐Gb3 analysis was collected approximately 24 h after the last sc dosing using sublingual plexus bleeding. Immediately afterwards, all animals were sacrificed, and the heart, kidney and liver were harvested, weighed and frozen at <−70°C.

### 2.7 Measurement of Gb3‐ and lyso‐Gb3‐concentrations

Gb3 in tissue and lyso‐Gb3 in serum samples were measured by liquid chromatography–tandem mass spectrometry (LC–MS/MS) as described before^21^ at pharm‐analyt Labor GmbH. Concentrations of Gb3 isoforms: Gb3‐C24‐0, Gb3‐C16‐0 and Gb3‐C18‐0 were calculated based on calibration curves spiked with respective reference standards. Both the reference standards and the internal standard used (Gb3‐C16‐0‐D_9_) were bought from Matreya. Gb3 results were reported as sum of 3 isoforms per gram of tissue.

### 2.8 Statistical analysis

Data are presented as mean ± SD. Statistical significance was determined with one way ANOVA (Tukey's multiple comparison test), using GraphPad Prism (version 9.0).

## 3 RESULTS

### 3.1 PK and biodistribution study

**FIGURE 1:**
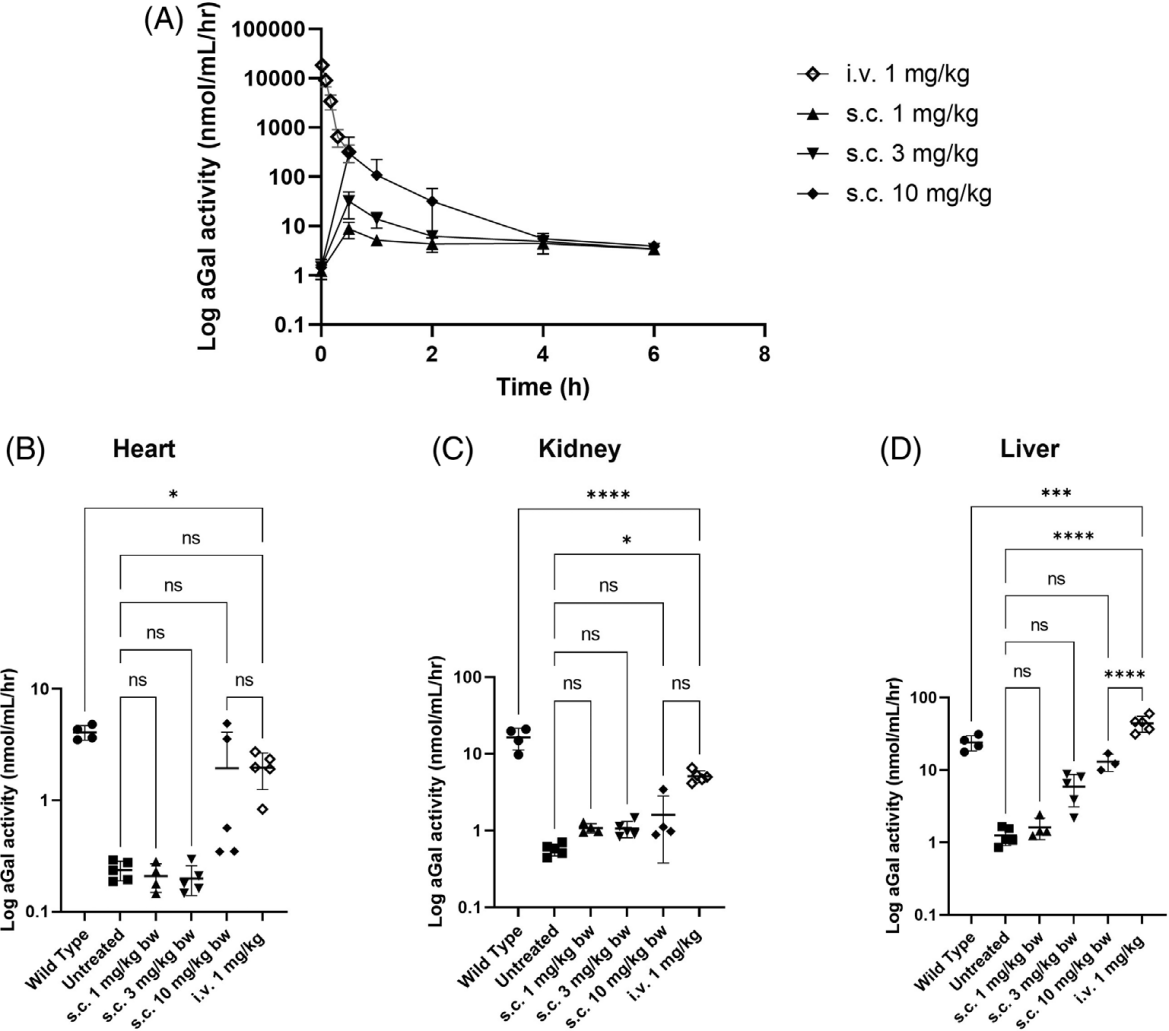
PK in plasma and biodistribution in tissues of WT and Fabry mice 24 h post sc (1,3,10 mg/kg bw) and iv (1 mg/kg bw) administration of moss‐aGal: (A) PK profile; aGal activity in (B) heart; (C) kidney, (D) liver. Data are presented as mean ± SD (*n* = 5) (*P < 0.05, **P < 0.01, ***P < 0.001, ****P < 0.0001). Only chosen statistical comparisons are shown.

The PK profile following iv administration was characterized by rapid clearance of moss‐aGal from circulation, with approximately 50% activity decrease within 5 min post injection. In contrast, sc application led to peak plasma activities at 0.5 h post injection, with enzyme activity returning to baseline at 4–6 h post injection. Peak plasma activities post sc injection were dose dependent and for the highest dose approx. 10^3^ lower than after iv application (Figure 1A). However, starting from 0.5 h post injection, plasma levels of the 10 mg/kg group were comparable to that of the iv treated animals.

Similarly, enzyme activities in organs 24 h post sc application were low with levels comparable to untreated Fabry animals for 1 and 3 mg/kg and markedly higher only for dose 10 mg/kg in the heart and liver (Figure 1B–D). In comparison, enzyme activities following iv injection in all examined organs were distinctly higher than that of untreated controls, only in liver surpassing the activity of the WT level (Figure 1B–D). In direct comparison between sc and iv applications, both lower sc doses 1 and 3 mg/kg achieved activities below 21% of the iv dose in all organs (Figure S1). The highest sc dose reached higher activities, with levels comparable to that of the iv injection only in the heart tissue.

Considering the half‐life of moss‐aGal in plasma, a shorter time span for tissue distribution read‐out seemed more appropriate. Timepoints: 2 h‐post iv and 4 h‐post sc injections were chosen for the follow‐up biodistribution study. However, these results did not differ from those obtained 24 h post injection (Figure S2).

### 3.2 Cellular localization study

**FIGURE 2:**
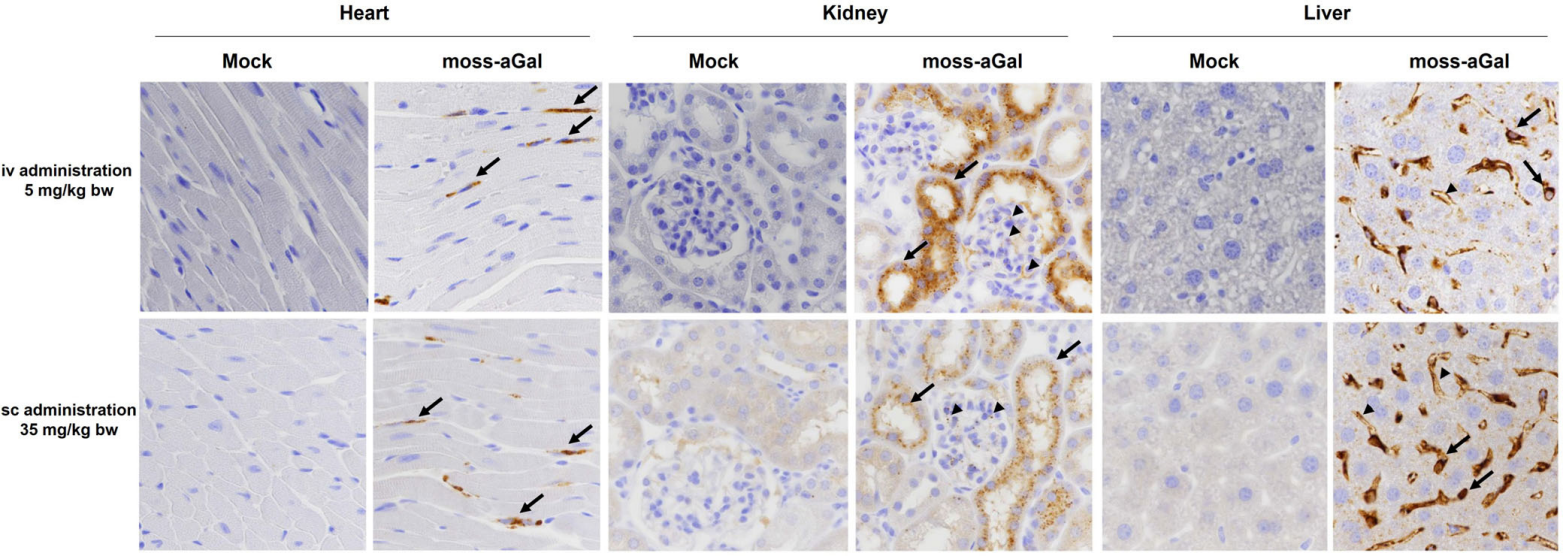
Cellular localization of moss‐aGal in the heart, kidney and liver following an iv (5 mg/kg bw) and sc (35 mg/kg bw) administration.

Independent of the route of administration, the cellular localization of moss‐aGal in the heart, kidney, and liver of Fabry mice was comparable (Figure 2). In the heart, the enzyme was detected in microvascular endothelial cells and interstitial fibroblasts (arrows). In kidneys, specific signals were distributed in cortical tubules (arrows) and in glomeruli (arrowheads). Positive signals in the liver were in endothelial cells (arrowheads) and putative Kupffer cells (arrows).

### 3.3 Comparison of efficacy between repeated sc and iv applications of moss‐aGal

In the initial study, efficacy of Gb3 reduction in organs of Fabry mice was evaluated following eight subcutaneous injections of moss‐aGal (at 1; 3 and 10 mg/kg) compared to vehicle treated control. Eight injections led to a significant, dose dependent decrease in Gb3 levels in heart and liver and lyso‐Gb3 levels in mouse serum (Figure S3A,C,D) with best results achieved for 10 mg/kg. In contrast, the reduction of Gb3 deposits in the kidneys was less pronounced (Figure S3B), with only highest dose achieving markedly lower Gb3 levels than the control group.

**FIGURE 3:**
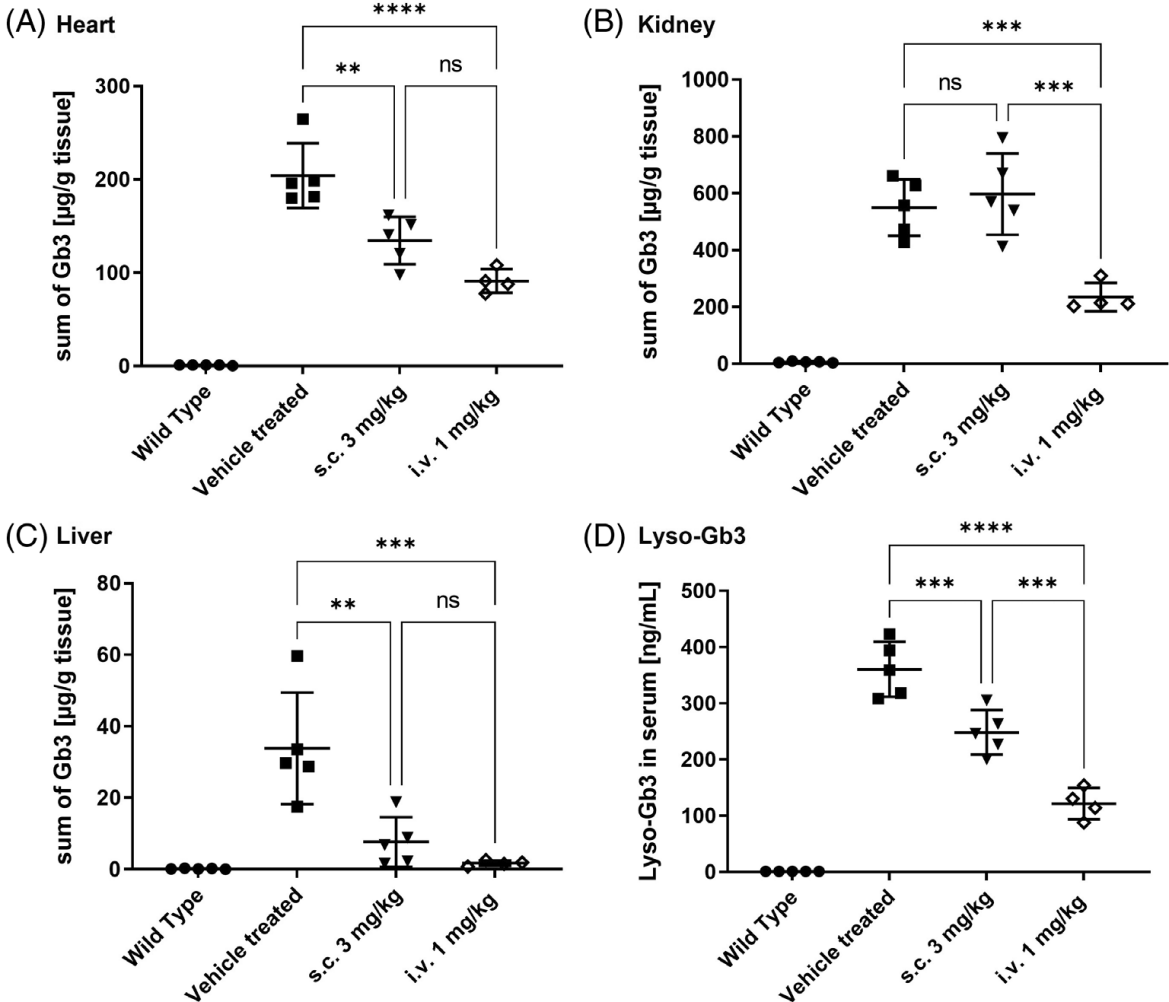
Gb3 content in tissues of WT and Fabry mice following 3× iv (1 mg/kg bw) or 16× sc administrations of moss‐aGal (3 mg/kg bw) or vehicle: (A) heart, (B) kidney, (C) liver, (D) Lyso‐Gb3 concentration in mouse serum. Data are presented as mean ± SD (*n* = 4–5) (*P < 0.05, **P < 0.01, ***P < 0.001, ****P < 0.0001). Only chosen statistical comparisons are shown.

A follow‐up study aiming to compare the efficacy of repeated, prolonged sc treatment (at 3 mg/kg, 16 injections) with an iv administration according to a protocol of current standard of care for agalsidase beta (1 mg/kg, biweekly) was performed. In good agreement with the results from the initial study, a significant decrease of Gb3 concentrations in the heart and liver, as well as in serum lyso‐Gb3 levels was observed (Figure 3A,C,D). However, treatment prolongation did not improve the Gb3‐reduction in the kidneys (Figure 3B). In all measured tissues and serum samples, the efficacy of 3 iv injections was clearly higher than that of 16 sc injections (Figure 3). In summary, prolongation of the sc treatment did not result in better efficacy. In contrast, three iv injections with a dose of 1 mg/kg led to better Gb3 clearance in all tissues, especially in the kidney (Figure S4).

## 4 DISCUSSION

The results described in the present study provide evidence that, the subcutaneously applied moss‐aGal reaches target organs and can degrade Gb3 deposits in tissues of Fabry mice. However, in direct comparison to the iv administration, it is less efficient. An obvious reason for the lower efficacy of sc injection is an insufficient bioavailability following this route of administration (Figure S1). Sc delivery rarely reaches the bioavailability of iv administration and often higher doses are required to compensate for this drawback.^18^ While intravenously‐administered biotherapeutic is placed directly into the systemic circulation (100% bioavailability), an injection into the extracellular space of the sc tissue requires transport from the injection site to the circulation through a mixture of vascular and lymphatic absorption.^15^, ^22^ While the data from the cellular localization study support the notion that moss‐aGal arrives at the same cells independently of administration route (Figure 2), the absolute amounts of enzyme reaching the organs differ considerably. As shown in Figures 1B–D and S1 even with higher doses of 3 and 10 mg/kg lower or comparable enzyme activities were reached in target organs following sc injection compared to the iv (1 mg/kg) injection. Notably, the tissue concentration of aGal required to achieve good Gb3 clearance appears to be organ specific. While enzyme activities are lower in the heart than in the kidney after sc injection (11% vs. 21%, Figure S1), the corresponding Gb3 reduction is more efficient in this organ (33% vs. 8%, Figure S4). This observation is in line with reports by others indicating that Gb3 clearance is least effective in the kidneys of Fabry mice.^23^, ^24^

Use of higher dose could increase moss‐aGal's bioavailability (Figure S1). Results from the initial efficacy study show dose dependency; with the strongest Gb3 reduction obtained for 10 mg/kg (Figure S3), consistent with the highest enzyme activities for this dose. However, more frequent administration of 10× dose of the iv‐infusion is economically not feasible—a reason for selecting the middle dose (3 mg/kg) for the comparability efficacy study. In view of these findings, it would be interesting to examine the possibility of an initial use of high iv dosages for clearance of bulk of Gb3 deposits, followed by sc‐treatment, delivering low but constant enzyme levels, which might be enough to prevent the recurrence of Gb3 build‐ups.

Another aspect of bioavailability is related to the drug's formulation. In our studies, we used a formulation, developed for an iv application. Since factors such as composition, pH, temperature, viscosity, and tonicity of the dosing formulation are known to influence drug's bioavailability in sc delivery,^15^ fine tuning of the moss‐aGal formulation could significantly improve its bioavailability. A switch from isotonic to hypertonic buffer with addition of O‐phosphoserine, dramatically enhanced lymphatic uptake of monoclonal antibody—Rituximab and increased its bioavailability from 29% to 99%.^22^

Further limitation of our study concerns the use of a mouse model. Morphological and physiological comparison between rodent and human skin reveals differences and significant variability in bioavailability between these species for diverse molecules has been described.^16^ For instance, a PEGylated Fab fragment of a monoclonal antibody—Certulizumab, has shown a modest bioavailability (24%–34%) in rats and considerable bioavailability in humans (76%–88%).^25^

Taken together, our results demonstrate that a sc application of moss‐aGal leads to favorable degradation of Gb3 deposits in Fabry mouse model and thus suggest the possibility of using this route of administration for therapeutic treatment of FD. To enable clinical application, targeted adaptation of the formulation to increase bioavailability and further studies examining the optimal dose and application scheme are warranted.

## AUTHOR CONTRIBUTIONS

**Paulina Dabrowska‐Schlepp**: conceptualization; data curation; formal analysis; visualization; project administration; writing – original draft; writing – review and editing. **Andreas Busch**: conceptualization; data curation; project administration; writing – review and editing. **Jin‐Song Shen**: methodology; investigation; resources; formal analysis; writing – review and editing. **Rachel Y. Cheong**: investigation; resources; writing – review and editing. **Lone Bruhn Madsen**: resources; writing – review and editing. **Daniel Mascher**: methodology; investigation; formal analysis; writing – review and editing. **Raphael Schiffmann**: conceptualization; methodology; writing – review and editing. **Andreas Schaaf**: conceptualization; data curation; funding acquisition; writing – review and editing.

## FUNDING INFORMATION

All studies, which results are presented in the following article, were financially supported by Eleva GmbH. Paulina Dabrowska‐Schlepp, Andreas Busch and Andreas Schaaf are employees of Eleva GmbH and are co‐authors of a patent for moss‐aGal (European patent: EP16714275.1A).

## CONFLICT OF INTEREST STATEMENT

The authors declare no conflict of interest.

## ANIMAL WELFARE

All institutional and national guidelines for the care and use of laboratory animals were followed.

In vivo efficacy studies performed at Timeline Bioresearch AB (now Scantox Sweden, Lund) were conducted in accordance with ethical permit number 5.8.18–18 164/2021 of Swedish Animal Experiments Inspectorate. In vivo experiments (PK, biodistribution and cellular localization study), which were performed at Baylor Research Institute (Texas, USA) were approved by the Institutional Animal Care and Use Committee of Baylor Research.

## Supporting information


**FIGURE S1:** Bioavailability of sc doses in organs of Fabry mice 24 h post sc (1, 3, 10 mg/kg bw) injection represented as % of activity after iv injection of moss‐aGal. Data are presented as mean ± SD (*n* = 5). Mean values are annotated above the bars.
**FIGURE S2:** Biodistribution in tissues of WT and Fabry mice 4 h post sc (1, 3, 10 mg/kg bw) and 2 h post iv (1 mg/kg bw) administration of moss‐aGal. aGal activity in (A) heart; (B) kidney and (C) liver. Data are presented as mean ± SD (*n* = 5) (*P < 0.05, **P < 0.01, ***P < 0.001, ****P < 0.0001). Only chosen statistical comparisons are shown.
**FIGURE S3:** Gb3 content in tissues of WT and Fabry mice following eight sc administrations of moss‐aGal (1, 3, 10 mg/kg bw) or vehicle: (A) heart, (B) kidney, (C) liver, (D) Lyso‐Gb3 concentration in mouse serum. Data are presented as mean ± SD (*n* = 5) (*P < 0.05, **P < 0.01, ***P < 0.001, ****P < 0.0001). Only chosen statistical comparisons are shown.
**FIGURE S4:** Comparison of moss‐aGal efficacy in clearing accumulated Gb3 in tissues between 8 and 16 sc injections (3 mg/kg bw) and 3 iv (1 mg/kg bw) injections. Efficacy is reported as Gb3% vehicle treated. Statistical significance was determined with two‐way ANOVA and Tukey's multiple comparison test. Data are presented as mean ± SD (*n* = 4–5) (*P < 0.05, **P < 0.01, ***P < 0.001, ****P < 0.0001). All statistical comparisons are shown.

## Data Availability

The data that support the findings of this study are available from the corresponding author upon reasonable request.
