# Supplementary material for: Comparison of efficacy between subcutaneous and intravenous application of moss‐aGal in the mouse model of Fabry disease
Source: JIMD Rep. 2023 Sep 12;64(6):460–7. doi: 10.1002/jmd2.12393 (PMC10623099; doi:10.1002/jmd2.12393)
Supplement: Supplementary file 1 — FIGURE S1: Bioavailability of sc doses in organs of Fabry mice 24 h post sc (1, 3, 10 mg/kg bw) injection represented as % of activity after iv injection of moss‐aGal. Data are presented as mean ± SD (n = 5). Mean values are annotated above the bars. FIGURE S2: Biodistribution in tissues of WT and Fabry mice 4 h post sc (1, 3, 10 mg/kg bw) and 2 h post iv (1 mg/kg bw) administration of moss‐aGal. aGal activity in (A) heart; (B) kidney and (C) liver. Data are presented as mean ± SD (n = 5) (*P < 0.05, **P < 0.01, ***P < 0.001, ****P < 0.0001). Only chosen statistical comparisons are shown. FIGURE S3: Gb3 content in tissues of WT and Fabry mice following eight sc administrations of moss‐aGal (1, 3, 10 mg/kg bw) or vehicle: (A) heart, (B) kidney, (C) liver, (D) Lyso‐Gb3 concentration in mouse serum. Data are presented as mean ± SD (n = 5) (*P < 0.05, **P < 0.01, ***P < 0.001, ****P < 0.0001). Only chosen statistical comparisons are shown. FIGURE S4: Comparison of moss‐aGal efficacy in clearing accumulated Gb3 in tissues between 8 and 16 sc injections (3 mg/kg bw) and 3 iv (1 mg/kg bw) injections. Efficacy is reported as Gb3% vehicle treated. Statistical significance was determined with two‐way ANOVA and Tukey's multiple comparison test. Data are presented as mean ± SD (n = 4–5) (*P < 0.05, **P < 0.01, ***P < 0.001, ****P < 0.0001). All statistical comparisons are shown. [file JMD2-64-460-s001.docx]

**Supplementary material**

**Figure S1**


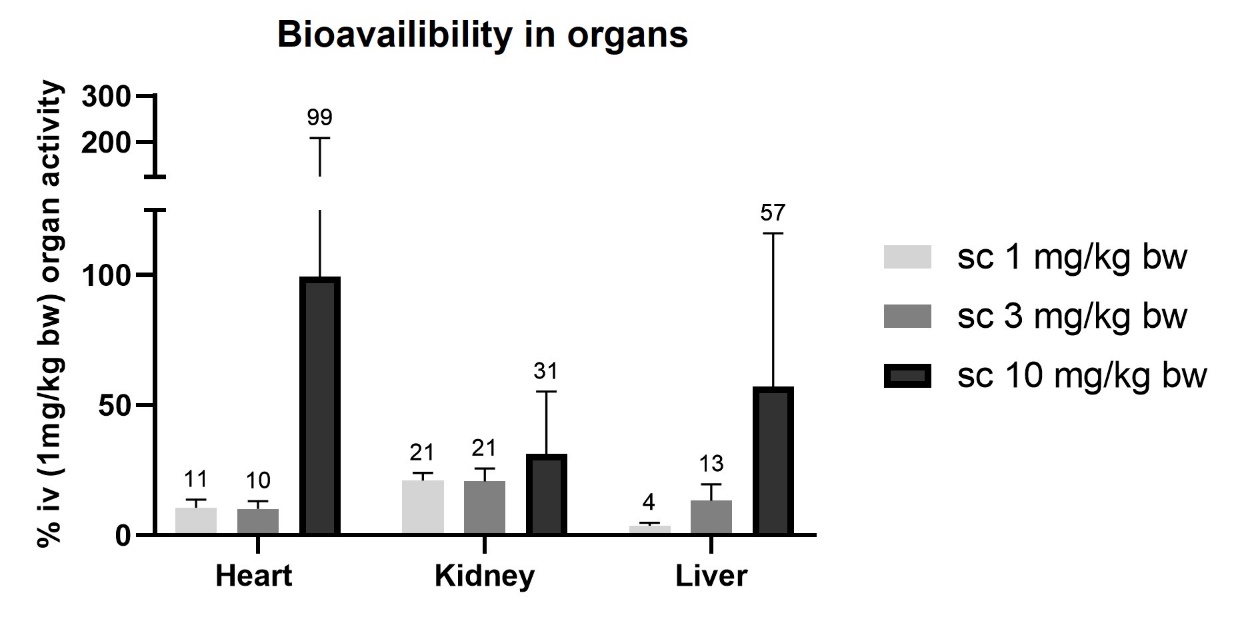


**Figure S1:** Bioavailability of sc doses in organs of Fabry mice 24 hours post sc (1,3,10 mg/kg bw) injection represented as % of activity after iv injection of moss-aGal. Data are presented as mean±SD (n=5). Mean values are annotated above the bars.

**Figure S2**


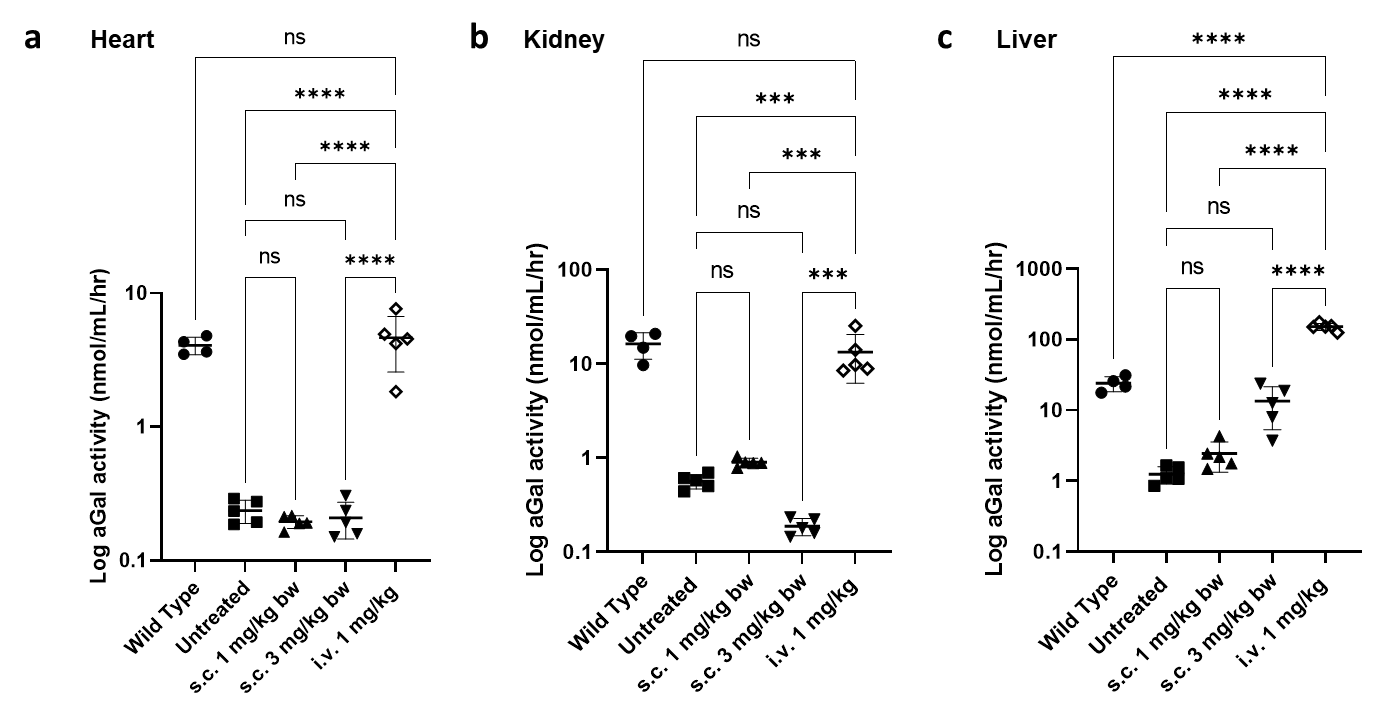


**Figure S2**: Biodistribution in tissues of WT and Fabry mice 4h post sc (1,3,10 mg/kg bw) and 2h post iv (1 mg/kg bw) administration of moss-aGal. aGal activity in a. heart; b. kidney and c. liver. Data are presented as mean±SD (n=5) (*P<0.05,**P<0.01, ***P<0.001, ****P<0.0001). Only chosen statistical comparisons are shown.

**Figure S3**


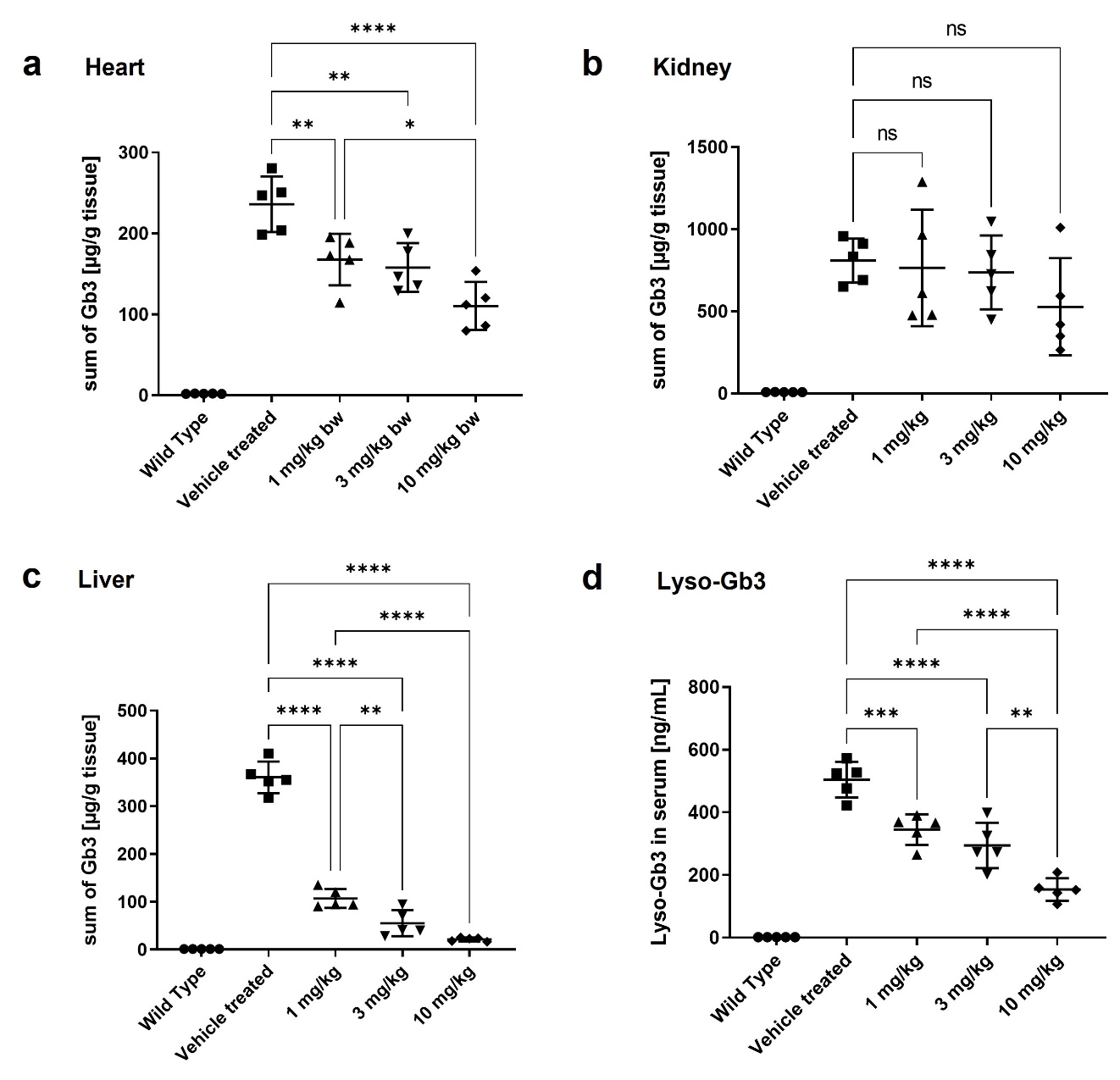


**Figure S3:** Gb3 content in tissues of WT and Fabry mice following 8 sc administrations of moss-aGal (1,3,10 mg/kg bw) or vehicle: a. heart, b. kidney, c. liver, d. Lyso-Gb3 concentration in mouse serum. Data are presented as mean±SD (n=5) (*P<0.05, **P<0.01, ***P<0.001, ****P<0.0001). Only chosen statistical comparisons are shown.

**Figure S4**

**Figure S4:** Comparison of moss-aGal efficacy in clearing accumulated Gb3 in tissues between 8 and 16 sc injections (3 mg/kg bw) and 3 iv (1 mg/kg bw) injections. Efficacy is reported as Gb3 % vehicle treated. Statistical significance was determined with two-way ANOVA and Tukey’s multiple comparison test. Data are presented as mean±SD (n=4-5) (*P<0.05, **P<0.01, ***P<0.001, ****P<0.0001). All statistical comparisons are shown.
